# Supplementary material for: Modeling glioblastoma heterogeneity as a dynamic network of cell states
Source: Mol Syst Biol. 2021 Sep 16;17(9):e10105. doi: 10.15252/msb.202010105 (PMC8444284; doi:10.15252/msb.202010105)
Supplement: Supplementary file 5 — Source Data for Figure 3 [file MSB-17-e10105-s001.zip › Figure3A_sourcedata/GSEA_3065/hallmarks_state5.GseaPreranked.1623416302988/HALLMARK_INFLAMMATORY_RESPONSE.html]

Details for gene set HALLMARK\_INFLAMMATORY\_RESPONSE[GSEA]

|  || Dataset | state5 |
| Phenotype | NoPhenotypeAvailable |
| Upregulated in class | na\_pos |
| GeneSet | HALLMARK\_INFLAMMATORY\_RESPONSE |
| Enrichment Score (ES) | 0.42906943 |
| Normalized Enrichment Score (NES) | 1.6370105 |
| Nominal p-value | 0.0042918455 |
| FDR q-value | 0.029455375 |
| FWER p-Value | 0.053 |
Table: GSEA Results Summary

  

Fig 1: Enrichment plot: HALLMARK\_INFLAMMATORY\_RESPONSE      
 Profile of the Running ES Score & Positions of GeneSet Members on the Rank Ordered List

  

| PROBE | GENE SYMBOL | GENE\_TITLE | RANK IN GENE LIST | RANK METRIC SCORE | RUNNING ES | CORE ENRICHMENT || 1 | TIMP1 |  |  | 109 | 0.518 | 0.0315 | Yes |
| 2 | ITGB8 |  |  | 126 | 0.503 | 0.0722 | Yes |
| 3 | SELENOS |  |  | 197 | 0.441 | 0.1016 | Yes |
| 4 | ATP2A2 |  |  | 212 | 0.431 | 0.1364 | Yes |
| 5 | EMP3 |  |  | 264 | 0.400 | 0.1645 | Yes |
| 6 | TNFAIP6 |  |  | 380 | 0.349 | 0.1810 | Yes |
| 7 | LDLR |  |  | 404 | 0.339 | 0.2070 | Yes |
| 8 | HAS2 |  |  | 462 | 0.322 | 0.2277 | Yes |
| 9 | P2RX7 |  |  | 473 | 0.319 | 0.2535 | Yes |
| 10 | SLC4A4 |  |  | 598 | 0.286 | 0.2637 | Yes |
| 11 | SLC11A2 |  |  | 601 | 0.285 | 0.2876 | Yes |
| 12 | IFNAR1 |  |  | 614 | 0.282 | 0.3100 | Yes |
| 13 | PDPN |  |  | 719 | 0.258 | 0.3201 | Yes |
| 14 | DCBLD2 |  |  | 863 | 0.230 | 0.3235 | Yes |
| 15 | PTPRE |  |  | 893 | 0.227 | 0.3394 | Yes |
| 16 | ATP2B1 |  |  | 953 | 0.218 | 0.3512 | Yes |
| 17 | ATP2C1 |  |  | 957 | 0.217 | 0.3692 | Yes |
| 18 | PDE4B |  |  | 968 | 0.215 | 0.3862 | Yes |
| 19 | TAPBP |  |  | 1047 | 0.204 | 0.3947 | Yes |
| 20 | PSEN1 |  |  | 1217 | 0.183 | 0.3911 | Yes |
| 21 | SERPINE1 |  |  | 1221 | 0.182 | 0.4062 | Yes |
| 22 | RAF1 |  |  | 1275 | 0.175 | 0.4150 | Yes |
| 23 | SLC31A1 |  |  | 1371 | 0.164 | 0.4182 | Yes |
| 24 | AXL |  |  | 1495 | 0.153 | 0.4173 | Yes |
| 25 | OSMR |  |  | 1571 | 0.144 | 0.4211 | Yes |
| 26 | GABBR1 |  |  | 1652 | 0.138 | 0.4237 | Yes |
| 27 | CX3CL1 |  |  | 1776 | 0.128 | 0.4207 | Yes |
| 28 | MET |  |  | 1992 | 0.112 | 0.4060 | Yes |
| 29 | CALCRL |  |  | 2109 | 0.105 | 0.4019 | Yes |
| 30 | ABCA1 |  |  | 2142 | 0.103 | 0.4069 | Yes |
| 31 | CD82 |  |  | 2145 | 0.102 | 0.4153 | Yes |
| 32 | BTG2 |  |  | 2174 | 0.100 | 0.4206 | Yes |
| 33 | KCNJ2 |  |  | 2175 | 0.100 | 0.4291 | Yes |
| 34 | GNAI3 |  |  | 2504 | 0.079 | 0.3989 | No |
| 35 | HRH1 |  |  | 2507 | 0.079 | 0.4053 | No |
| 36 | SLC7A1 |  |  | 2527 | 0.077 | 0.4097 | No |
| 37 | ADORA2B |  |  | 2563 | 0.076 | 0.4122 | No |
| 38 | CSF1 |  |  | 2643 | 0.071 | 0.4093 | No |
| 39 | ACVR2A |  |  | 2674 | 0.070 | 0.4119 | No |
| 40 | PLAUR |  |  | 2826 | 0.061 | 0.4001 | No |
| 41 | IRF1 |  |  | 2926 | 0.056 | 0.3937 | No |
| 42 | ITGA5 |  |  | 3140 | 0.045 | 0.3736 | No |
| 43 | KLF6 |  |  | 3447 | 0.029 | 0.3418 | No |
| 44 | TNFRSF1B |  |  | 3727 | 0.019 | 0.3121 | No |
| 45 | KIF1B |  |  | 3759 | 0.017 | 0.3101 | No |
| 46 | BST2 |  |  | 3916 | 0.011 | 0.2935 | No |
| 47 | TPBG |  |  | 4075 | 0.004 | 0.2761 | No |
| 48 | NDP |  |  | 4442 | -0.012 | 0.2361 | No |
| 49 | MYC |  |  | 4656 | -0.020 | 0.2139 | No |
| 50 | NFKBIA |  |  | 4840 | -0.027 | 0.1956 | No |
| 51 | NFKB1 |  |  | 5183 | -0.039 | 0.1606 | No |
| 52 | SCN1B |  |  | 5371 | -0.048 | 0.1436 | No |
| 53 | ABI1 |  |  | 5375 | -0.048 | 0.1473 | No |
| 54 | NMI |  |  | 5523 | -0.054 | 0.1354 | No |
| 55 | IRF7 |  |  | 5524 | -0.054 | 0.1400 | No |
| 56 | RIPK2 |  |  | 5661 | -0.060 | 0.1298 | No |
| 57 | LY6E |  |  | 5890 | -0.069 | 0.1101 | No |
| 58 | EIF2AK2 |  |  | 6481 | -0.100 | 0.0524 | No |
| 59 | RGS16 |  |  | 6543 | -0.104 | 0.0543 | No |
| 60 | CHST2 |  |  | 6560 | -0.106 | 0.0615 | No |
| 61 | AHR |  |  | 6678 | -0.112 | 0.0578 | No |
| 62 | IFNGR2 |  |  | 6865 | -0.127 | 0.0477 | No |
| 63 | HIF1A |  |  | 6996 | -0.137 | 0.0446 | No |
| 64 | NAMPT |  |  | 7250 | -0.162 | 0.0299 | No |
| 65 | RELA |  |  | 7298 | -0.167 | 0.0387 | No |
| 66 | RHOG |  |  | 7585 | -0.202 | 0.0237 | No |
| 67 | ADRM1 |  |  | 8548 | -0.458 | -0.0456 | No |
| 68 | CDKN1A |  |  | 8679 | -0.545 | -0.0142 | No |
| 69 | SRI |  |  | 8709 | -0.572 | 0.0308 | No |
Table: GSEA details [plain text format]

  

Fig 2: HALLMARK\_INFLAMMATORY\_RESPONSE: Random ES distribution      
 Gene set null distribution of ES for **HALLMARK\_INFLAMMATORY\_RESPONSE**
